# Understanding different aspects of blood supply of uterine fibroids: an overview of ultrasound and magnetic resonance imaging techniques

**DOI:** 10.1186/s13244-025-02033-2

**Published:** 2025-09-16

**Authors:** Derk Jan Slotman, Marissa Frijlingh, L. J. M. Juffermans, L. W. Bartels, C. T. W. Moonen, T. Bosch van den, M. F. Boomsma, J. A. F. Huirne

**Affiliations:** 1 Department of Radiology, Isala, Zwolle, The Netherlands; 2 Image Sciences Institute, Imaging & Oncology Division, University Medical Center Utrecht, Utrecht, The Netherlands; 3 Department of Obstetrics and Gynecology, Amsterdam UMC, Amsterdam, The Netherlands; 4 Amsterdam Reproduction and Development, Amsterdam, The Netherlands; 5 Focused Ultrasound Foundation, Charlottesville, Virginia USA; 6 Department of Obstetrics and Gynecology, University Hospital Leuven, Leuven, Belgium; 7 Department of Development and Regeneration, KU Leuven, Leuven, Belgium

**Keywords:** Uterine fibroid, Medical imaging, Blood circulation, Regional blood flow

## Abstract

**Background:**

Vasculature and blood flow play a crucial role in the genesis, diagnosis, and treatment of uterine fibroids. Assessment of the different aspects of blood flow by medical imaging has therefore gained a prominent place in the clinical management and research of uterine fibroids. Imaging is most often performed with ultrasound and MRI. However, consistent terminology describing the blood flow and vasculature of fibroids is lacking, impeding efficient interdisciplinary communication.

**Purpose:**

This narrative review provides information on blood flow and vasculature in uterine fibroids and offers insights into ultrasound and MRI techniques for measuring and visualizing these physiological parameters. To contribute to consistent terminology, we propose a generic nomenclature for different aspects of blood flow and vasculature.

**Discussion:**

Fibroids generally have a complex and variable vascular composition, which can be unraveled for a large part with the wide range of currently available ultrasound and MRI techniques. Future studies focusing on uterine fibroids may benefit from the connections laid in this article between fibroid vasculature, qualitative and quantitative ultrasound and MRI techniques and our proposed nomenclature.

**Critical relevance statement:**

This review aims to contribute to a deeper understanding of the blood supply of uterine fibroids and the available imaging techniques for its visualization. This may lead to improved clinical management of uterine fibroids and more efficient interdisciplinary communication.

**Key Points:**

- Blood supply is crucial in the genesis, diagnosis, and treatment of fibroids.
- Ultrasound and MRI can unravel the complex vascular composition of fibroids.
- Consistent nomenclature around blood supply can improve clinical management of fibroids.

**Graphical Abstract:**

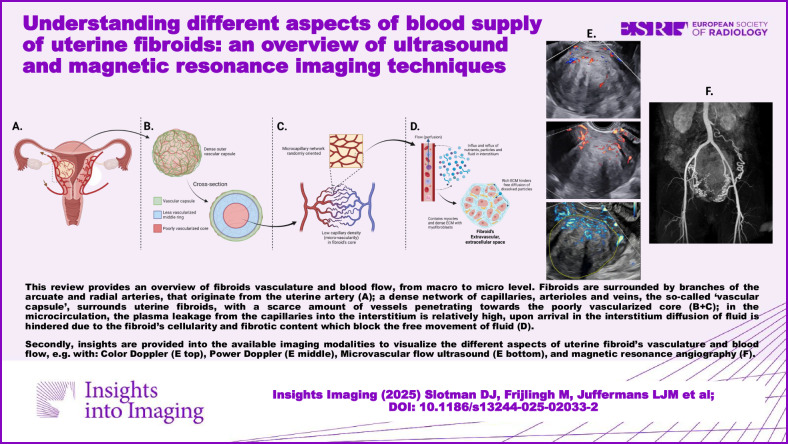

## Introduction

Uterine fibroids are benign smooth muscle cell tumors, also referred to as (leio)myomas [1, 2]. They are the most common gynecological tumors with a cumulative incidence of more than 70% at the age of 50 [3]. A woman can have one or multiple fibroids, which can be located throughout the uterus, either close to the endometrium (submucosal), within the uterine wall (intramural), or on the outside of the uterus and bulging into the abdominal cavity (subserosal) [4]. Symptoms are abnormal uterine bleeding, pelvic pain, bulk-related symptoms or infertility [5], leading to more than 200,000 hysterectomies per year in the United States due to fibroids [6]. Besides surgical options, less invasive alternative treatments have become available [7]. In both surgical and non-surgical treatments of uterine fibroids, blood flow plays an important role in screening for eligibility, intra-procedural monitoring, prognosis, and follow-up [8–22].

Already at the stage of initial diagnosis, assessment of blood flow may be crucial to discriminate fibroids from adenomyosis or sarcomas [20, 23]. Misdiagnosis of adenomyosis as a fibroid can result in failure to identify the cleavage plane and increased risk of complications when treated minimally invasively (e.g., myomectomy) [24, 25]. In unexpected sarcomas, myomectomy may influence the prognosis due to intraoperative tumor spill [26]. In addition, outcomes after minimally invasive treatments such as embolization and ablation are correlated with the blood flow of a fibroid [10–19]. Also in disease prognosis, including severity of symptoms, fertility and quality-of-life, blood flow plays an important role, e.g., fibroids with increased blood flow have a higher growth rate compared to uterine fibroids with low blood flow [8, 9, 27, 28]. Accordingly, assessment of the different aspects of blood flow has gained a prominent place in the management of uterine fibroids over the last 20 years.

Imaging of a uterine fibroid’s blood flow-related aspects is commonly performed with ultrasound or magnetic resonance imaging (MRI) techniques. However, consistent terminology and definitions of what exactly is imaged using either ultrasound or MRI are lacking. Consistent terminology is of great importance to avoid misunderstanding between radiologists and referring physicians [29]. Both types of disciplines could profit from an overview of the different aspects of fibroid blood flow and of the imaging modalities suitable to measure or visualize such aspects, in both clinical and research settings. As ultrasound is often interpreted by referring physicians and MRI by radiologists, consistent nomenclature could facilitate reporting and efficient inter-specialty communication, and help with the selection of the most suitable diagnostic modality. In some institutions, full imaging scans are only available to the specialty acquiring the diagnostic imaging, leaving other specialties dependent on the imaging reports. In these situations, clear terminology is especially of great importance.

The aim of this work is (1) to propose a consistent nomenclature related to blood supply, (2) to provide detailed information on uterine fibroids’ blood supply using this consistent nomenclature, and (3) to describe different imaging modalities suitable for assessing the fibroids’ vasculature, blood flow and tissue microstructure.

## Blood flow, transvascular transport, and diffusion

The circulation of blood aims to perfuse and drain different organs and tissues. Perfusion can generally be defined as the intravascular flow of blood per unit tissue mass [30, 31]. See Table 1 for definitions and proposed consistent nomenclature. The blood circulation can be divided into an afferent (arterial) and efferent (venous) part. Based on criteria related to vessel size, the circulation can be further divided into two major structural compartments: macro- and microvasculature. The macrovasculature (Table 1) is the anatomical network of large, medium-sized and small arteries and veins (diameters down to 0.1 mm) that form a transportation network for blood. The microvasculature (Table 1) is defined as the terminal network of vascular structures inside organs. The boundary between micro- and macrovasculature remains arbitrary to some degree, and varies in literature between 20 and 500 µm [32–34]. Within the microvasculature, arterioles (10–500 µm) are responsible for blood flow distribution, capillaries (5–10 µm) for molecular blood-tissue exchange, and venules (10–500 µm) for drainage of the venous blood [32].

**Table 1:** As a wide range of definitions of blood flow and vascularity-related expressions are used in both clinical practice and research, we propose this nomenclature to contribute to consistent terminology

Term	Definition
Vasculature	Anatomic network of vascular structures
Microvasculature	Vascular structures with a diameter ≤ 20 µm (e.g., arterioles, capillaries, venules)
Macrovasculature	Vascular structures with a diameter > 20 µm (e.g., arteries, veins)
Vascularization	The process of growing a vascular network into a tissue
Vascularity	The degree to which a vascular network is present in tissue
Circulation	Intravascular flow of blood from and toward the heart driven by the cardiac output
Microcirculation	Circulation in the microvasculature
Macrocirculation	Circulation in the microvasculature
Perfusion	Intravascular flow of blood (either in micro- or macrovasculature) per unit tissue mass
Diffusion	Net movement of a substance from high to low concentration, as a result of the random erratic movement of particles in a liquid (or gas). Often referred to as the motion of particles from the intravascular space to the interstitium and vice versa
Permeability	Characteristic of a vascular structure referring to the leakage of liquid, plasma proteins and solutes through endothelial junctions (predominantly in the microvasculature)

In the capillaries, molecules leave the intravascular space by passive, active, and vesicular transport mechanisms to the surrounding tissue. Molecular displacement from plasma across the endothelial barrier is driven by the hydrostatic pressure and depends on the permeability (Table 1) of capillaries and bulk flow (the movement of fluid down a pressure gradient). Upon arrival in the extracellular extravascular space, interstitial fluid continues moving by diffusion and bulk flow [35]. Diffusion (Table 1) can be defined as the motion of dissolved particles in a liquid or gas from high to low concentration (Fick’s law) as a net result of ‘Brownian motion’, i.e., the random motion of particles in a fluid. Diffusion is an example of a passive transport mechanism. The velocity and direction of the dissolved particles (e.g., nutrients, oxygen, proteins, metabolic waste) in interstitial fluid depend on bulk flow and diffusion, which is influenced by the architecture of the extracellular matrix in terms of collagen, cell density, and microvascular density [36, 37].

## The vasculature of uterine fibroids

**Fig. 1:**
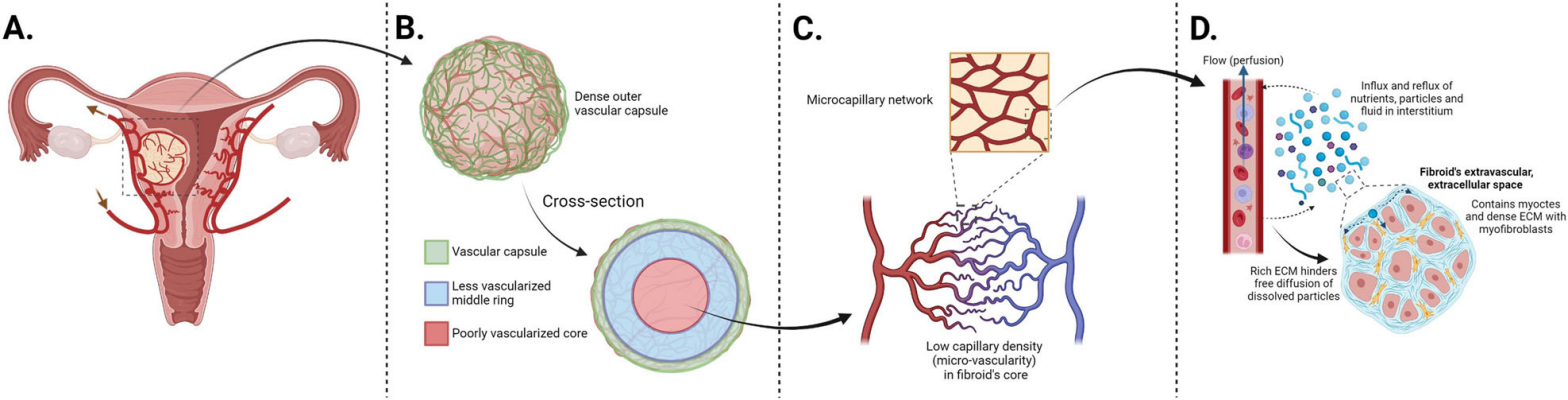
Overview of fibroids vasculature, from macro- to microcirculation: fibroids are surrounded by branches of the arcuate and radial arteries, that originate from the uterine artery (**A**); a dense network of capillaries, arterioles and veins, the so-called ‘vascular capsule’, surrounds uterine fibroids, with a scarce number of vessels penetrating toward the poorly vascularized core (**B**, **C**); in the microcirculation, the plasma leakage from the capillaries into the interstitium is relatively high, upon arrival in the interstitium diffusion of fluid is hindered due to the fibroid’s cellularity and fibrotic content which block the free movement of fluid (**D**). ECM, extracellular matrix

The uterine vasculature, which is described in detail by Ciarmela et al [38], is relevant for the growth of uterine fibroids. The normal macrovasculature primarily comprises two uterine arteries, originating from the left and right internal iliac arteries, which are also called the hypogastric arteries. The uterine artery runs bilaterally to the outer surface of the uterus [38]. Both uterine arteries branch into superficial and inferior arteries that end in circumferential arcuate arteries in the myometrium [38–41]. Branches from the arcuate and subsequent radial arteries continue as microvascular structures surrounding the uterine fibroid and eventually penetrating the fibroid center (Fig. 1A, B). The vascular structures and density of the microvasculature depend on the size and location of the fibroid [42, 43].

When a fibroid is growing, it compresses the surrounding myometrium. Fibroids < 3 mm are encircled mostly by capillaries and a few arterioles and veins. Although this surrounding network is relatively dense compared to the myometrium, an extremely dense vascular network is seen in fibroids > 1 cm. This so-called vascular capsule consists mainly of arterioles, capillaries and venules, as part of both the macro- and microvasculature (Fig. 1B). The macrovasculature of this vascular capsule is larger in diameter compared to the macrovasculature of the normal myometrium, also the microvasculature is more dense [44–48]. The few arteries that the ‘vascular capsule’ contains either penetrate directly toward the center or originate from the periphery of the fibroid. The arteries penetrating directly to the center have few side branches, while arteries originating from the periphery branch quite quickly into arterioles and capillaries [42]. The center itself is only slightly vascularized and has no intrinsically structured vascular arrangement [48–51]. Uterine fibroids receive blood via vessels expanded from the myometrial macro- and microvasculature [48].

However, before oxygen and nutrients reach the fibroid’s myocytes, they need to traverse a relatively large distance from the capillaries, especially in the fibroid’s center, where the vascular density is low [52]. In a fibroid, the average distance from capillaries to myocytes ranges between 35 and 58 μm, depending on fibroid subtype, compared to only 20–30 μm in healthy tissue [36]. As mentioned earlier, vascular permeability plays a role in molecular displacement from plasma across the endothelial barrier. This permeability depends on the maturity of the capillaries [44, 53] and on the presence of pericytes and vascular smooth muscle cells [46, 54]. It is up for discussion whether the flux from the intravascular compartment to the extravascular space within fibroid tissue is limited by the hydrostatic pressure or by the capillary permeability (Figs. 1D, 6).

Angiogenesis, the growth of new blood vessels from pre-existing capillaries, enables fibroids to grow and has an important role in a fibroid’s main vascular characteristics, such as perfusion rate, permeability and diffusion. The role of angiogenesis in uterine disorders has been extensively discussed by Harmsen et al [55] and Middelkoop and Don et al [46, 55]. On one hand, a relatively mature vasculature and low permeability have been reported, based on the benign character of fibroids [15, 56]. On the other hand, fibroids often outgrow their demand for nutrients and oxygen, which is accompanied by incomplete angiogenesis, resulting in a permeable neovasculature with increased capillary leakage [15, 57]. When a fibroid outgrows its blood supply, degeneration starts. The resulting shortage of oxygen can lead to different types of degeneration, such as hyaline, myxomatous, calcific, cystic, fatty, red (usually only during pregnancy), or necrotic [58, 59].

The vascular anatomy described above and the interstitial space supplying the uterus and uterine fibroids, can be depicted by different imaging techniques, which will be discussed in the following paragraphs.

## Imaging

A range of imaging modalities are available for the visualization of fibroid’s vasculature, blood flow, and mobility of water molecules in the extravascular space. To adequately utilize these imaging modalities, it is pivotal to weigh in the subtle differences in the meaning of terms such as vascularization, perfusion and diffusion (Table 1), as well as the physics underlying the imaging principles.

As ultrasound and MRI are the most commonly applied imaging modalities in fibroids, their available options will be outlined in this section and Table 2 for the visualization and measurement of a fibroid’s vasculature, blood flow, and tissue microstructure.

**Table 2:** The relation between imaging modalities (MRI and ultrasound) and the different levels of a uterine fibroid’s blood supply





In this table, the typical goal of the imaging techniques is described, and the macro-/microcirculatory components are marked in which underlying physiological processes take primarily place. Imaging techniques were selected that have been described in the uterine fibroid literature

** Requires an exogenous contrast agent

## Ultrasonography

**Fig. 2:**
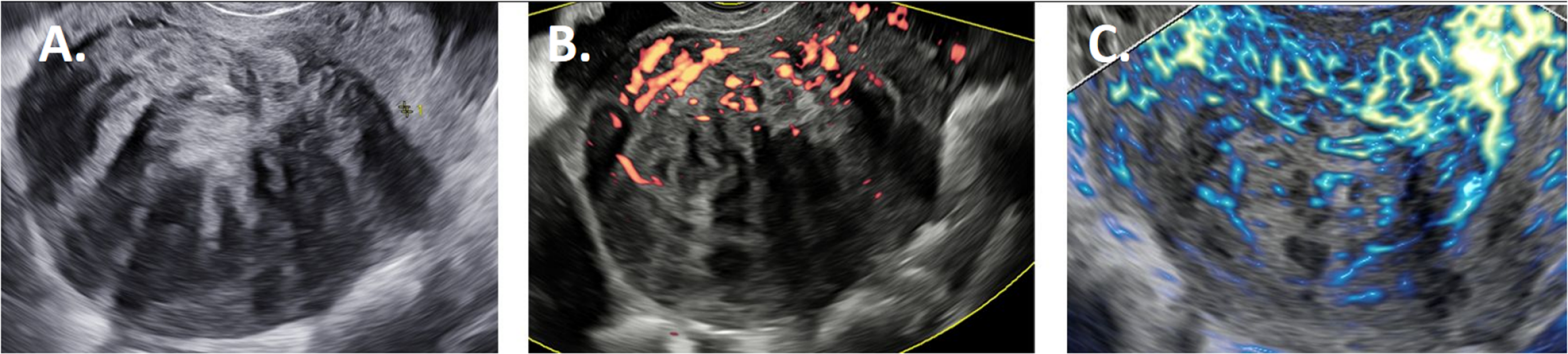
Sagittal fibroid images obtained by the different ultrasound techniques in patient 1. 2D B-mode ultrasound (**A**); 2D Power Doppler (**B**); 2D Microvascular flow ultrasound (**C**)

**Fig. 3:**
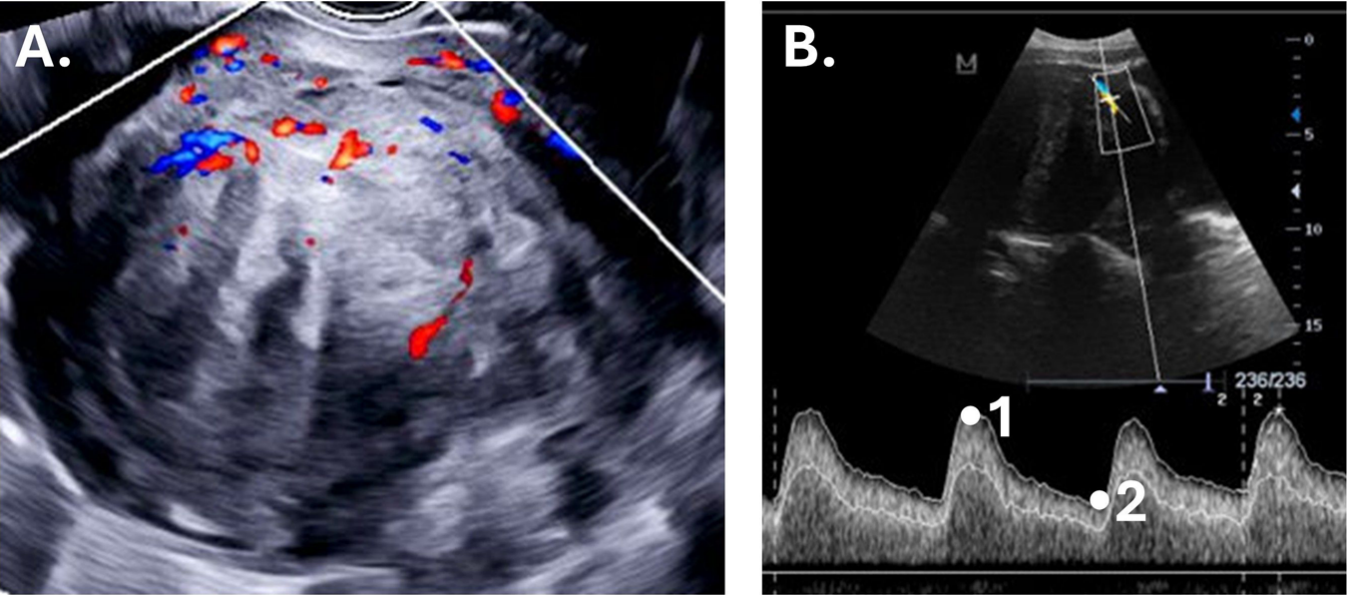
Sagittal fibroid images from patient 2 obtained by 2D Color Doppler ultrasound (**A**), including color hues reflecting location and direction of blood flow; and 2D Spectral Doppler ultrasound (**B**) from patient 3 [80], including the gate to detect Doppler shift and showing blood flow velocity (*y*-axis) plotted against time (*x*-axis), resulting in a peak systolic velocity (**1**) and end diastolic velocity (**2**)

**Fig. 4:**
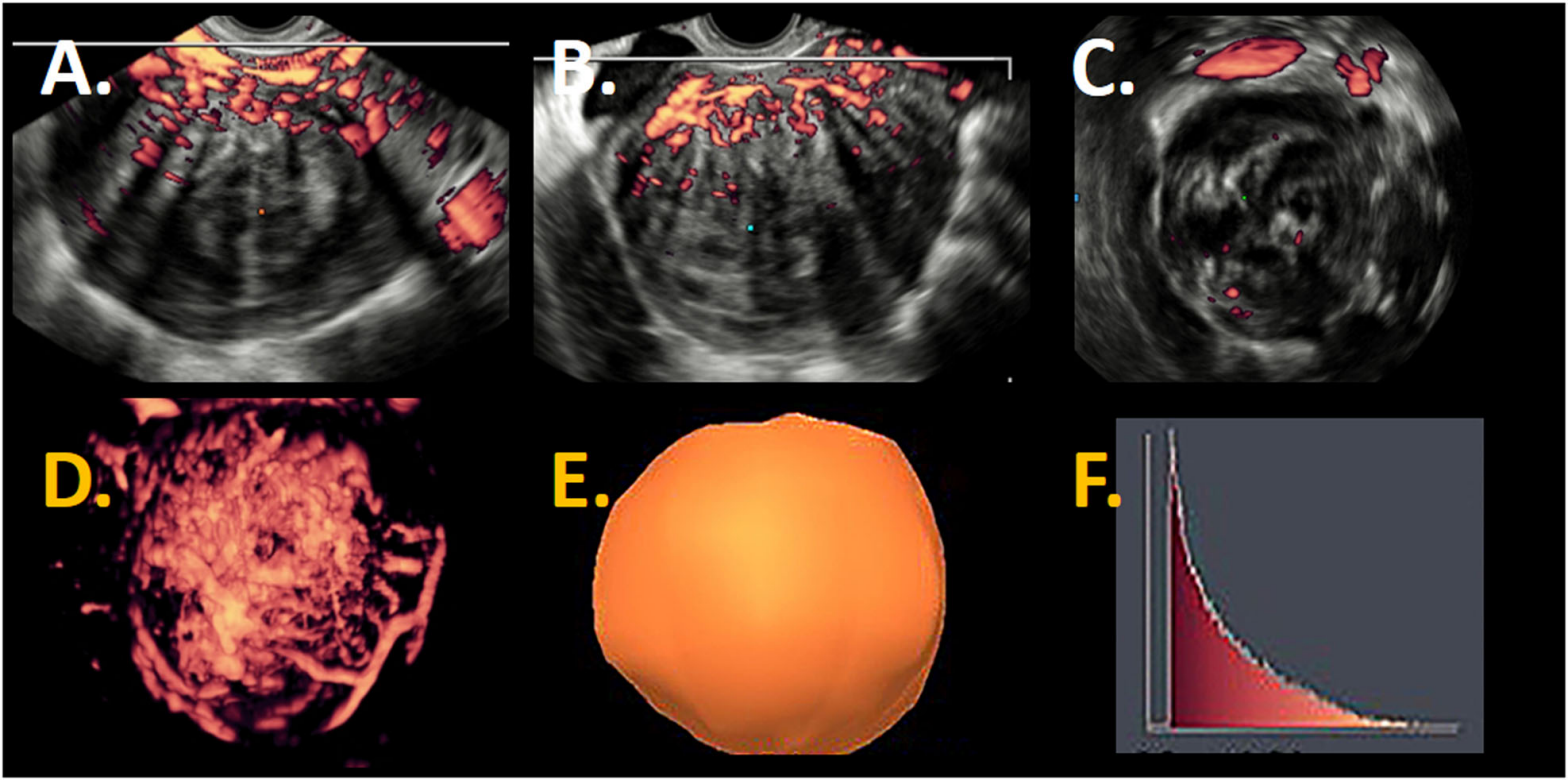
Overview 3D power Doppler images in patient 1. In sagittal view (**A**), transversal view (**B**), and coronal view (**C**). 3D power Doppler reconstruction (**D**) with a 3D power Doppler volume (**E**) as result and a histogram (**F**) as output of 3D power Doppler ultrasound with vascular indices as result

**Fig. 5:**
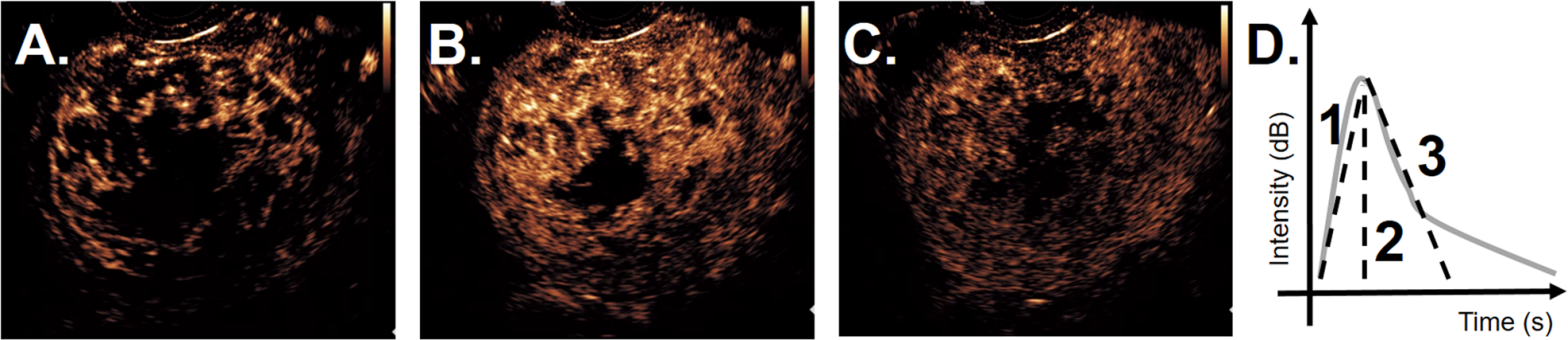
Contrast-enhanced ultrasound images of patient 1 at different time points after injection of contrast agents showing fibroid perfusion, t = 28 s (**A**); t = 32 s (**B**); t = 100 s (**C**). Quantification of the contrast signal results in a time-intensity curve (**D**), from which parameters such as wash-in rate (**1**), peak intensity (**2**) and wash-out rate (**3**) can be obtained

Ultrasound offers a widely available, dynamic method to visualize fibroids. Figures 2–5 give an overview of fibroid images obtained by the different ultrasound techniques described in the following paragraphs. Preferably, a transvaginal probe is used because of the higher frequency (5–7.5 MHz) than an abdominal probe (around 3.5 MHz), providing higher resolution images [20, 60]. In daily clinical practice, the ultrasound examination is standardly started with 2D B-mode scans.

### Ultrasound—B-mode

The standard ultrasound imaging mode is called Brightness Mode (B-mode, Fig. 2A). It is based on visualization of reflection of longitudinal ultrasound waves from interfaces between tissues with different acoustic impedances [58, 61].

B-mode images are 2D grayscale images displaying tissue anatomy and structure, which are constructed by the ultrasound machine from reflected and scattered ultrasound waves emitted and received by multiple piezoelectric crystals inside the transducer, which is placed on the skin of the patient. In B-mode, the brightness (gray values) of the pixels relates to the relative amplitude of reflected ultrasound waves. Using an estimation of the speed of sound propagation of 1540 m/s for human tissue, the location of the displayed pixel in the image is calculated by the difference in time between sending and receiving the ultrasound pulse. This average speed of sound is used by most diagnostic ultrasound systems. Speed variations caused by different tissues may result in marginal errors in size and shape. The errors are typically in the order of 2–5% [46, 49].

The reflected signal depends on the acoustic impedance (z) and relates to the tissue’s density (rho) and stiffness (*E*) by the formula *z* = $\sqrt{{rho}* E}$. The more density and stiffness differ between two types of tissue [46, 49], the higher the signal representing the reflection at their interface appears on B-mode images. In fibroids, the density and stiffness are influenced by the amount of collagen and smooth muscle cells, which are different for each individual fibroid [62].

B-mode ultrasound only visualizes secondary effects of perfusion [58, 59]. Blood scatters ultrasound waves at a very low level (typically 100–300 times less than tissue), while pure fluid doesn’t scatter ultrasound at all [63]. Therefore, blood vessels appear as hypo-echogenic, black structures on a B-mode image. Cysts, i.e., fluid-filled structures, can appear similar to hypo-echogenic lesions. To check whether hypo-echogenic structures are blood vessels or cysts, additional ultrasound techniques are required.

### Doppler ultrasound

Motion, such as blood flow, can be detected using Doppler ultrasound. A pulse of ultrasound waves is sent, and when reflected by a moving particle, such as erythrocytes in flowing blood, the frequency of the reflected beam changes with respect to that of the incoming wave. In case the movement of the particle is toward the probe, the wave is compressed, resulting in a higher frequency (positive Doppler shift), while motion away from the probe results in a lower frequency (negative Doppler shift). Sending and receiving of ultrasound waves can be done continuously (continuous wave Doppler) or intermittently (pulsed-wave Doppler) with a certain pulse repetition frequency (PRF). The advantage of pulsed-wave Doppler is the ability to calculate the location by dividing the pulse-echo travel time by twice the velocity [61].

#### Color and power Doppler ultrasound

Power Doppler and color Doppler show different aspects of flow and are normally superimposed on the grayscale image. For both modalities, a Doppler shift (change in frequency) has to be detected before any Doppler information is displayed. The difference between the two modalities is the way the Doppler information is processed.

In color Doppler (Fig. 3A), the magnitude and direction of the velocity of flow are displayed. The mean frequency shift for each area inside the selected region-of-interest is displayed as a color according to an arbitrary color code, representing the mean flow velocity. The color “red” is most often set by default to flow toward the transducer and blue away from the transducer. Differences in high and low velocities are displayed by different hues of red and blue. Laminar flow can be seen as a uniform color and turbulent flow as a mosaic pattern of all types of different color hues. Color Doppler is displayed on a grayscale image, thereby also providing the anatomical location of the macrovasculature. However, color Doppler is a delicate imaging technique, and the angle of insonation (theta), i.e., the position of the transducer with regard to the direction of the vessel, is pivotal for correct color Doppler imaging. In fact, the real Doppler velocity is ‘the measured Doppler velocity*cos (theta)’. So when the angle is 90°, blood flow is perpendicular to the ultrasound beam, and no Doppler shift can be detected (cos (90°) = 0). The Doppler shift is maximum when the insonation angle is near 0°, since cos (0°) = 1 [61]. An angle near 0°, however, is clinically not feasible, and an angle of 30–60° is sufficient.

Power Doppler displays only the magnitude of the Doppler signal, not the direction of flow, nor velocity (Fig. 2B). The magnitude (power) of the different Doppler frequency shifts in the region-of-interest is summed together to form the total power Doppler signal and is displayed as a color. The higher this power Doppler signal, the brighter the color. The power Doppler signal from each point in the region-of-interest represents the number of moving erythrocytes, i.e., the total amount of blood moving [64].

Power Doppler has a higher sensitivity for depicting vessels located inside organs than color Doppler, and is especially suitable to display blood vessels with slow flow in fibroids [65, 66]. Power Doppler is less sensitive to the angle of insonation than color Doppler and is not affected by aliasing. However, it is confined to imaging macrovascular structures larger than 0.1 mm [67, 68].

Imaging fibroids with Doppler ultrasound, the typical vascular capsule (Fig. 2) is clearly visible [38, 69, 70]. In daily clinical practice, the level of blood flow in a fibroid can easily be semi-quantified by a subjective color score, which is based on the power Doppler color hue in combination with an estimate of the percentage of vascularized tissue. Typically, the center of a fibroid has a color score of ‘1’ (no flow) or ‘2’ (minimal flow), and color score ‘3’ (moderate flow) or ‘4’ (abundant flow) at the site of the capsule [20].

#### Three-dimensional Doppler ultrasound

Three-dimensional (3D) Doppler ultrasound requires a separate 3D probe, which creates 3D-images by concatenating 2D-images along a third axis by mechanical rotation of the probe (Fig. 4). The added value of 3D-Doppler ultrasound compared to 2D is B-mode imaging of the coronal plane and providing a structural impression of the vascular architecture in 3D. The level of blood supply can be objectively determined by calculating the following parameters: Vascularity Index (VI), Flow Index (FI) and Vascularization Flow Index (FVI) in a region-of-interest. These are internationally defined parameters and used by multiple ultrasound systems. VI denotes the ratio of color-coded voxels (by power Doppler) and total voxels within the volume of interest and is expressed as a percentage. VI is an indication of the percentage of tissue that contains detectable blood flow. FI represents the mean power Doppler signal intensity from all color-coded voxels. Vascularization flow index (VFI) reflects mean flow velocity; VFI = VI*FI [20, 71]. Studies have shown that imaging a fibroid’s blood supply using 3D power Doppler is reliable and correlates well with the vascular density determined in regions-of-interest on histology [72, 73].

#### Spectral Doppler ultrasound

In spectral Doppler, the Doppler shift is detected within a measurement area defined by two parallel lines (the gate) and plotted against time (Fig. 3B). The spectral Doppler plot is displayed below or aside the grayscale image. Besides blood flow velocity, the waveform of the plot also provides clinical information [41, 64, 74]. Spectral Doppler parameters can be quantitatively measured and can be used to determine indices referring to the resistance and compliance of the blood vessels (Table 2). Spectral Doppler is generally used to display the cardiac cycle within an artery, i.e., in fetal imaging, to determine blood flow in the umbilical cord or cerebral artery. There is no general application of spectral Doppler in gynecology; the parameters are mentioned in a number of studies regarding fibroid symptoms and treatments [28, 38, 75–79]. Spectral Doppler parameter values seem to be lower in fibroids compared to normal myometrium and compared to fibroids with necrosis and secondary degeneration [74, 80, 81]. Outcomes might also vary with respect to either measuring the leading vessel, such as the uterine artery, or a blood vessel of the fibroid itself.

#### Microvascular flow imaging

A Doppler technique with advanced machine settings is microvascular flow imaging (Fig. 2C). Microvascular flow (MV-flow) imaging aims at imaging low blood flow velocities in small vessels by suppressing noise and small tissue movements without removing the weak signal originating from slow flow [58]. Compared to conventional power Doppler, the higher sensitivity is the result of optimized ultrasound machine settings, being a low PRF, a high frame rate and advanced motion filters. The low PRF allows detection of lower velocities, since detectable velocity = distance*PRF, where the distance is the difference between the depth of the target at two consecutive pulses [61]. More time in between pulses also results in a higher penetration. To prevent all low-frequency movement from being displayed as blood flow, advanced motion filters are needed. Microvascular flow imaging also shows the Doppler shift of each moving particle as color pixels superimposed on the B-mode image. As with power Doppler, it allows semi-quantitative measurements. The vascularity index (%) is calculated by dividing the number of colored pixels by the total number of pixels (grayscale + colored pixels) in a region-of-interest. Although only a limited number of studies are published on microvascular flow imaging, this technique indeed seems to be able to depict small vessels with slow blood flow in fibroids [16, 58, 82].

### Contrast-enhanced ultrasound

Although MV-flow imaging may already provide a view of the microvasculature, depicting the microvasculature in detail is dedicated to contrast-enhanced ultrasonography (CEUS, Fig. 5). Ultrasound contrast agents consist of encapsulated gas-filled microbubbles with a diameter ranging between 0.5 and 8 µm, allowing them to pass all capillaries following intravenous injection. The large difference in acoustic impedance between the gas core and the surrounding tissues results in the reflection of a strong backscatter signal [83]. Once exposed to the ultrasound wave, the microbubbles start oscillating due to the alternating positive and negative pressures of the ultrasound wave, thereby reflecting the ultrasound even stronger than merely based on acoustic impedance. On top of that, this oscillation of the microbubble is non-linear, so not only is the insonifying frequency reflected, but also the multiples of the insonifying frequency. Modern ultrasound machines have a so-called ‘contrast mode’ where these harmonics are received, creating a unique image showing only the microbubbles without the tissue (tissue does not show non-linear reflections). This is an accurate method for showing the blood pool and vascular structures [84]. As these microbubbles are true intravascular agents, i.e., they do not extravasate into the interstitium, they reflect true perfusion of an organ and/or lesion. Another advantage of this technique is that CEUS images can be fully quantified via so-called time-intensity curves. From these graphs, blood perfusion parameters, such as peak intensity, wash-in rate, time-to-peak, and wash-out rate, can be extracted. These parameters are organ-specific and may help to differentiate between benign disorders (fibroids) and malignancies (sarcomas) in the future [84–86].

## Magnetic resonance imaging

Magnetic resonance imaging (MRI) plays an important role as a second-stage imaging technique in the diagnosis and treatment-guidance of uterine fibroids, as it allows comprehensive imaging of the uterus and uterine masses [87]. MRI offers methods for radiation-free acquisition of 2D and 3D datasets with a high spatial resolution [88], with typical voxel sizes in the range of millimeters, but even finer sub-millimeter voxel sizes are possible [89]. A wide range of MRI techniques is available for the visualization of the uterine fibroid’s vasculature, blood flow, and tissue microstructure (Table 2). A distinction can be made between techniques that require and those that do not require an exogenous contrast agent, which is denoted in Table 2.

### T1- and T2-weighted imaging (Table 2A, B)

**Fig. 6:**
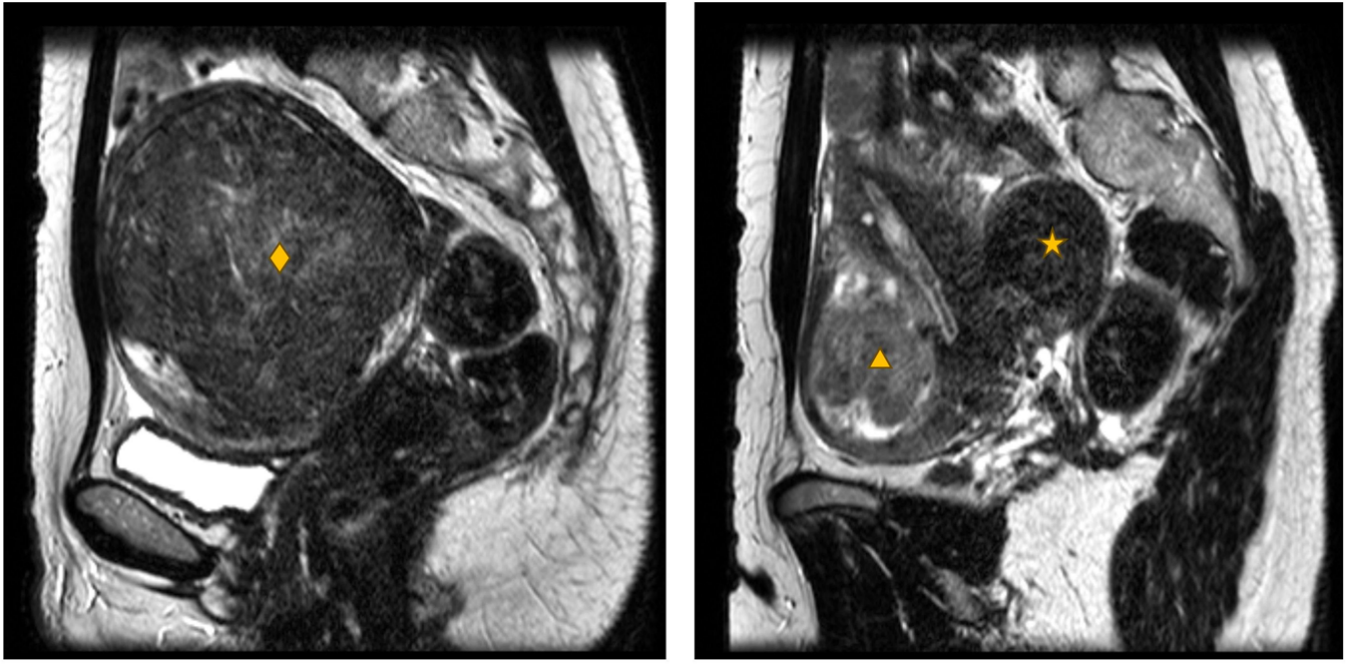
Sagittal T2w images of a 45-year-old patient with a large uterus containing multiple uterine fibroids. Left: intramural uterine fibroid with medium T2w signal intensity in between myometrium and skeletal muscle (Funaki type 2), with a volume of 406 cm^3^ (diamond). Right: uterine fibroid in contact with endometrium with high T2w signal intensity comparable to myometrium (Funaki type 3), with a volume of 57 cm^3^ (triangle), and subserosal Funaki type 2 uterine fibroid with a volume of 36 cm^3^ (star)

The clinical core of diagnostic MRI consists of proton-density-weighted (PDw), T1-weighted (T1w), and T2-weighted (T2w) imaging to visualize anatomy and pathology. Several aspects of the vasculature of fibroids can be clearly visualized using T1w and T2w-MRI, including the vascular capsule and degeneration as a consequence of deficient blood flow (Fig. 6) [90–92]. It is important to note that the grayscale images of T1w and T2w scans do not represent signal measurements on an absolute scale, but rather show relative contrasts with mixed contributions from multiple tissue characteristics. As a consequence, T1w and T2w scans do not allow quantitative assessment and comparison of signal intensity. Alternatively, quantitative T1- and T2-parameter maps can be calculated using T1- and T2-mapping techniques (Table 2C), but require additional scans that are typically time-consuming. It has been shown that T2-mapping can be a potential tool to quantify common uterine lesions, including different types of fibroids, and distinguish benign from malignant uterine lesions [93, 94]. The T2 value can be used to study tissue composition and reflect the water content of tissue. Verpalen et al [93, 94] found that T2-mapping contributed to the discrimination of fibroid tissue types and proposed that quantitative MRI may be a useful tool to determine the optimal treatment modality. Zhu et al [93, 94] found that the T2 values of benign tumors were significantly lower than those of malignant tumors. Fibroids showed particularly low T2 values, which is the probable result of the proliferation of smooth muscle cells. T2-mapping reflects the relative presence of water, but not in which micro-compartment the water resides (interstitium, cell, vascular structure).

By calculating scaled signal intensities (SSIs) from T1w and T2w scans using a reference structure, signal intensities can be analyzed in a semi-quantitative manner. A similar approach has been relatively successfully applied in the context of MR-guided high-intensity focused ultrasound (MR-HIFU) treatments of fibroids (as per Funaki scale [95]) by comparing T2w signal intensities of fibroids to those in myometrium and skeletal muscle. The resulting metric (Funaki scale) is used to estimate the degree to which fibroid tissue can be heated [94, 96–99].

### Contrast-enhanced MRI (Table 2D)

**Fig. 7:**
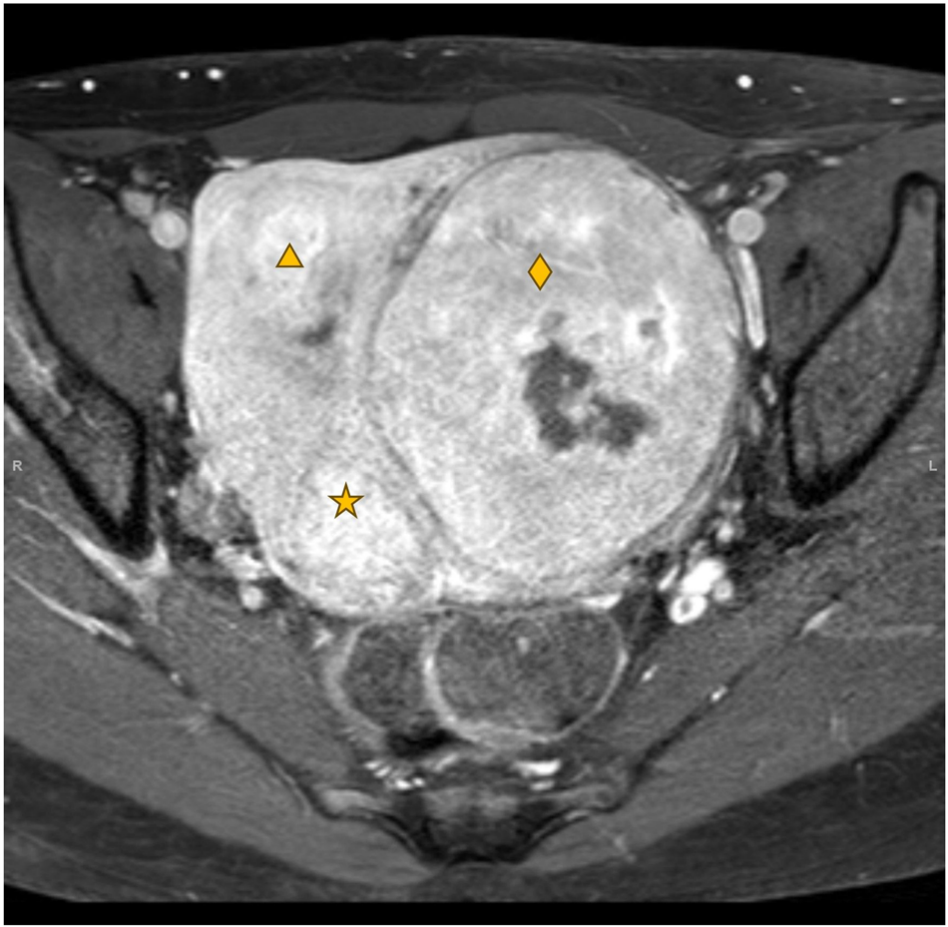
Axial contrast-enhanced T1w scan of the same patient depicted in Fig. 6 during the same scanning session. The largest fibroid on the left (diamond) has a central non-enhancing component with absence of contrast uptake, consistent with degeneration. This is a common appearance in large uterine fibroids, as they often outgrow their poor central vascularization

Contrast-enhanced (CE) MRI is another commonly used technique that can be applied to further assess fibroids’ vasculature and blood flow, by injection of a contrast medium typically combined with a T1-weighted sequence. Contrast agent molecules, often Gadolinium-based, shorten T1-relaxation time of the blood or tissue they reside in, resulting in a brighter appearance of areas where contrast molecules are present (Fig. 7). After the arterial phase, the uterine fibroid’s interstitium enhances based on extravasation of the contrast agent. The rate of extravasation depends on the contrast agent, vascular surface area, permeability and arterial input [100, 101].

CE-T1w is a useful tool to identify the type of fibroid degeneration, which is often associated with low enhancement on CE-T1w scans. Additionally, CE-MRI is often used to assess the technical treatment outcome of uterine artery embolization (UAE) and HIFU treatments in uterine fibroids. Large non-perfused volumes in relation to the volume of the target fibroid are indicative of durable clinical success [102, 103]. A recent study by Liu et al [104] showed that one day after MR-HIFU treatment, the signal intensity ratio on CE-MRI between the untreated part of the fibroid and the myometrium is predictive for fibroid regrowth [104].

### Magnetic resonance angiography (MRA)/venography (MRV) (Table 2E–G)

**Fig. 8:**
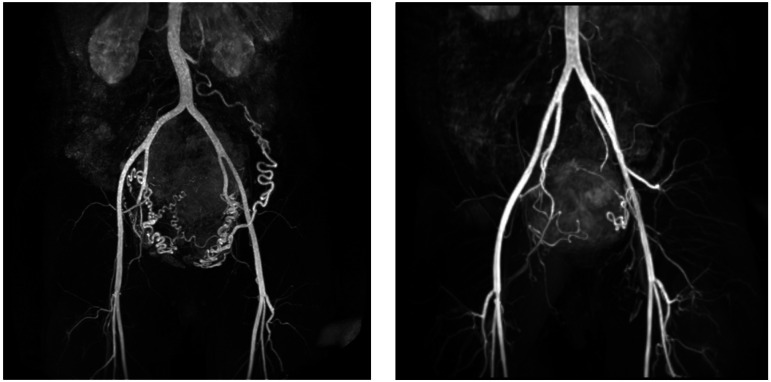
Magnetic resonance angiography (MRA) images of: subserosal/intramural uterine fibroid with a diameter of 144 mm in the dorsal uterine wall and hypertrophic arteria overica in a 41-year-old woman (left), and subserosal pedunculated fibroid of a 42-year-old woman with a 69 mm diameter (right)

Magnetic resonance angiography (MRA) can be used for the visualization of both vascular anatomy and blood flow (Fig. 8). Besides CE-MRA, a wide range of flow-based contrast agent-free MRA alternatives exists, most notably time-of-flight, black-blood, and phase-contrast techniques [105]. In regard to uterine fibroids, MRA is mainly used for screening before uterine artery embolization to assess geometric details of the pelvic vasculature [87, 106]. In addition, 4D-MRA (or time-resolved 3D phase-contrast MRA) offers a method for quantification of blood flow. A pilot study found a correlation between internal iliac flow measured by 4D-flow MRA and the ratio of embolic required for UAE of uterine fibroids [107].

In addition to MRA, MR venography (MRV, Table 2G) can be applied to evaluate venous anatomy using a contrast agent or flow-based MRI. Time-of-flight MRV may be used for investigating deep vein compression in large fibroids [108], or to detect changes in flow direction of ovarian veins caused by fibroid, and might help prevent the passive reflux into ovarian veins commonly seen in asymptomatic women [109].

### Dynamic contrast-enhanced (perfusion) MRI

Dynamic contrast-enhanced (DCE) MRI encompasses scanning multiple T1w images during the passage of the contrast agent over time. The goal of DCE-MRI is to gather information on hemodynamics and pharmacokinetics within an area-of-interest. DCE-MRI is often performed using a gadolinium-based contrast agent and is one of the main techniques for perfusion MRI [57, 101, 110–113].

**Fig. 9:**
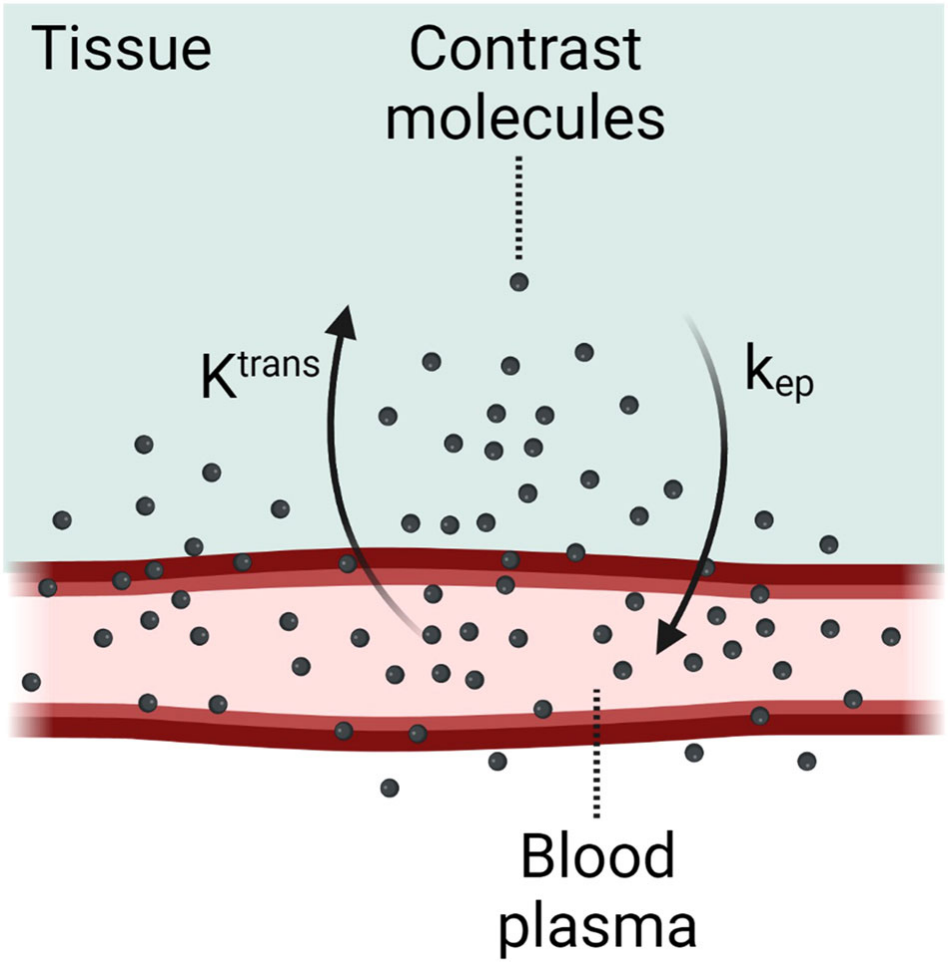
Tofts analysis of dynamic contrast-enhanced MRI (DCE-MRI) allows quantification of contrast agent transfer between the intracapillary space and interstitium. This includes the transfer rate from the capillary into the interstitium (K^trans^) and vice versa (k_ep_). Mathematical modeling (such as Tofts analysis) of DCE-MRI data can be useful when studying the vascular permeability within tissue

After venous administration, the contrast agent eventually arrives at the arterial end of the capillaries. There, the contrast molecules moves through the endothelial wall into the extravascular extracellular space due to their small molecular weight (Fig. 9). At the venous end of the capillaries, contrast molecules residing in the extravascular extracellular space return to the bloodstream.

Next to visual assessment of DCE-MRI, many metrics can be retrieved from the time-intensity curve within the tissue of interest, e.g., blood flow, blood volume, mean transit-time, and time-to-peak (Table 2H). For an extensive list of DCE-MRI parameters, see Gordon et al [110] or Khalifa et al [111].

In addition, information about the microcirculation (Table 1) can be derived by mathematical modeling [111]. By applying a pharmacokinetic model, typically Tofts [114] or Brix [115], to a concentration curve acquired with DCE-MRI, quantitative parameters related to vasculature and blood flow can be estimated. Parameters from Tofts modeling are listed in Table 2. With this modeling, several vascular characteristics can be observed, including intratumor angiogenic activity [101, 116]. The volume fraction of the extravascular extracellular space (v_e_) from Tofts modeling appears to be related to histological features of the fibroid, including degeneration and the amount of collagen. For instance, cystic fibroids have a higher v_e_-level [15].

DCE-MRI can be a useful tool to distinguish uterine fibroids from uterine sarcomas or ovarian fibroma [113, 117], or for the detection of vascular changes accompanying hormonal treatment [118]. Furthermore, DCE-MRI has been studied broadly for screening and evaluation purposes of HIFU treatments [15, 112, 119, 120].

### Arterial spin labeling (perfusion) MRI (Table 2I)

Another technique for perfusion MRI is arterial spin labeling (ASL). This method does not require an exogenous contrast agent, as it uses the blood itself as an endogenous contrast agent. With ASL, water protons in arterial blood are magnetically labeled using a radiofrequency pulse before they enter the region-of-interest. Subsequently, the arrival of the labeled blood in the region-of-interest is visualized. As the technique does not require an exogenous contrast agent, it provides a method for repeated non-invasive perfusion measurements, as opposed to DCE-MRI, wherein cumulative contrast agent exposure could be concerning. Within the context of uterine artery embolization, it has been shown that it is feasible to visualize the perfusion in fibroids and the response to uterine artery embolization [121]. In addition, ASL has been investigated in fibroid patients to assess myometrial perfusion, since fibroid-induced myometrial hypoperfusion is related to reduced fertility [121–123].

### Diffusion-weighted imaging (Table 2J)

With diffusion-weighted imaging (DWI), the random microscopic motion of water molecules due to diffusion can be measured and visualized. This includes measurements of the diffusivity in separate directions beyond (and in) the vasculature. Altered diffusivity in tissue can be indicative of various kinds of conditions, and may be used for tissue characterization, diagnosis, prognosis, and monitoring and prediction of treatment outcomes in fibroids [124, 125].

DWI is typically performed by acquiring a series of diffusion-weighted MR images with varying sensitivity to diffusion, but in some contexts, a DWI scan with a single sensitivity to diffusion suffices. The sensitivity to diffusion is characterized by the so-called ‘b-value’ (s/mm^2^), a parameter related to the strength and duration of the applied diffusion weighting magnetic field gradients.

**Fig. 10:**
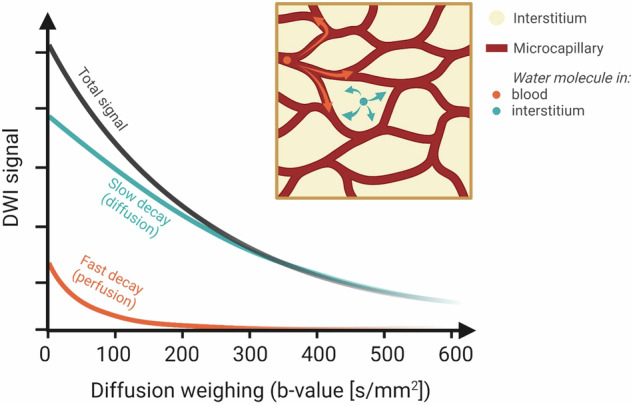
DWI is an MRI technique that enables visualization of the mobility of water molecules. By scanning with different sensitivities to random water mobility (expressed in b-values in s/mm^2^), the signal decay due to random water movement can be mapped. In biological tissue penetrated by capillaries, two noticeable effects are present, causing the signal to decay: diffusion and perfusion (or pseudo-diffusion). Diffusion is the result of Brownian motion, the random erratic movement of molecules in a fluid, and causes slow decay of DWI signal. The second noticeable component causing signal decay in DWI originates from water molecules in microcapillary blood flow (perfusion), and is often referred to as pseudo-diffusion. Due to the pseudo-random orientation, the microcapillaries cause a fast signal decay in DWI, typically in the range of 0–200 s/mm^2^. Using appropriate mathematical modeling, the fast and slow components can be separated from the DWI signal, allowing quantification of diffusion and perfusion in biological tissue

Diffusion of fluid in biological tissue occurs both within the intracellular and extracellular fluid and is rarely unhindered, but restricted instead by microscopic obstacles (e.g., cell membranes, organelles, fibers, proteins) (Fig. 1D). For this reason, diffusion restriction is higher in cellular fibroids, compared to degenerated fibroids [125]. DWI provides a method to measure the diffusion coefficient in a region-of-interest, the apparent diffusion coefficient (ADC). It is called apparent, as the coefficient obtained from DWI experiments does not necessarily reflect the true diffusion of water inside a single tissue compartment, but is also affected by other factors, such as scan parameter settings, tissue motion, and microcirculatory perfusion [126]. DWI may thereby reflect not only water molecule diffusivity but also microcirculatory tissue properties. This effect arises from the virtually random orientation of capillaries (Fig. 1C). Water molecules in capillary blood resemble Brownian motion, although with higher velocities, referred to as pseudo-diffusion. This may disturb the quantification of diffusion using ADC models. Pseudo-diffusion is characterized by a relatively fast decay compared to true diffusion; hence, it affects the DWI signal most noticeably in the low b-value range (< 200 s/mm^2^). By using more advanced DWI models, in which the pseudo-diffusion is incorporated, it is possible to separate microcirculatory flow (perfusion) effects from real diffusion (Fig. 10) [126–129].

An often used model for this purpose is the intravoxel incoherent motion (IVIM) model [126–130] that enables extraction of diffusion- and perfusion-related parameters from DWI images: diffusion coefficient *D*, pseudo-diffusion (perfusion) coefficient D*, and blood volume fraction *f*.

In the diagnosis and management of uterine fibroids, DWI can be exploited in various ways. DWI has been suggested as an optional scan by the European Society of Urogenital Radiology (ESUR) in MRI of fibroids [87]. ADC could play a role in differentiation between fibroids and malignant sarcomas and adenomyosis [87, 131–133], however its specificity has limitations [134–136]. Second, DWI has also been shown to be effective in the evaluation of fibroids before and after embolization and HIFU treatments [17, 137–142]. It can be used as a predictor of treatment outcomes [97, 141, 143], although more research is needed to clarify its exact role as a predictor of embolization outcomes [144].

## Discussion

In this review, we described the blood supply of uterine fibroids, proposed nomenclature (Table 1) and provided an overview of available ultrasound and MRI techniques for visualization of vasculature, blood flow, and tissue microstructure. Insights into what imaging can reveal of the vasculature, blood flow and tissue microstructure in uterine fibroids are important for understanding pathophysiology, diagnosis and for improving treatment outcomes of uterine fibroids. Second, to adequately utilize the ultrasound and MRI modalities by both radiologists and referring physicians in a clinical and research setting, consistent terminology around vasculature and blood flow is essential.

A comparable review is the work of Ciarmela et al [38], in which an overview is presented of fibroid’s vasculature from an anatomical, morphological and biomolecular point of view. Whereas our review focused on imaging from a gyneco-radiological point of view. Additionally, we emphasize the relevance of using consistent terminology and correct use of terms such as ‘vascularization’ (the process of growing a vascular network into a tissue) and ‘perfusion’ (intravascular flow of blood per unit tissue mass) (Table 1).

This review covered ultrasound and MRI, as they offer a wide range of imaging techniques that are complementary for imaging of the fibroid’s vasculature and blood flow. In clinical practice, ultrasound (B-mode) is typically applied as a first-line modality. Perfusion can be imaged using different Doppler techniques. In academic centers, advanced techniques such as MV-flow and CEUS imaging can additionally be applied to image blood flow in smaller blood vessels. CEUS is probably one of the most promising ultrasound techniques, being able to image the microvasculature in detail. As the contrast agents remain intravascular, CEUS provides quantitative information on perfusion. There is no ultrasound technique that can image diffusion.

MRI is mostly used as a second-line imaging modality in fibroids in case the ultrasound examination is not considered conclusive. This may occur in selective cases when doubts exist regarding the benign/malignant nature of the supposed fibroid, or when MRI is necessary to assess eligibility for minimally invasive (UAE) and non-invasive (MR-HIFU) treatments. MRI can be of added value because it provides tomographic 2D and 3D imaging with more anatomical details and superior soft-tissue contrasts when this is needed. Additional MRI techniques are available for imaging perfusion, e.g., CE-MRI and more advanced techniques including DCE-MRI and ASL. DWI is an interesting technique to visualize diffusion beyond (and in) the vasculature, and in addition, it offers a way to visualize capillary flow.

Other techniques that may also be helpful in visualization of fibroid’s vasculature and blood flow have so far not been mentioned in this work (including sono-elastography [145], ultrafast Doppler [146], dynamic 4D (time-resolved 3D) arterial spin labeling [107], computed tomography (CT) and digital subtraction angiography (DSA) [147]), as they have not been substantially studied in the context of uterine fibroids.

A limitation of our study is that we performed a non-systematic review regarding fibroid vasculature and blood flow, its nomenclature and suitable imaging. Information is biased due to the selection of relevant literature by the authors and their expert opinion. However, the research team consists of experts with experience in uterine imaging and treatment with various specializations, such as radiology, gynecology and medical physics. This design was chosen because of the relatively scarce amount of research data in the field of imaging of fibroid blood supply. Second, we presented a simplified overview of a uterine fibroid’s blood supply, while vascularization and blood flow depend on numerous factors, such as fibroid size, menstrual cycle, angiogenesis, and more. For a large part, we did not consider these factors when describing the imaging modalities.

This review may help in selecting the right imaging modality for future investigations of vascularization and blood flow-related topics around uterine fibroids. Clinical work and future fibroid studies focusing on angiogenesis, differentiation between benign/malignant, treatment selection or outcome prediction may benefit from uniform nomenclature as proposed in this work. In future efforts, the (advanced) imaging techniques as described in this review should be studied in order to improve individualized healthcare. This has the potential to eventually lead to improved conservative, pharmaceutical, non-invasive, minimally invasive and invasive surgical management of uterine fibroids.

In conclusion, using either ultrasound or MR imaging distinct aspects, such as micro- or macrovascular anatomy, perfusion and diffusion can be depicted, characterizing the complex vasculature and blood flow of uterine fibroids differently. To avoid confusion about what is shown or measured, it is pivotal to use consistent, cross-discipline nomenclature.

## Abbrevations

ADC: Apparent diffusion coefficient
ASL: Arterial spin labeling
B-mode: Brightness mode
CE: Contrast-enhanced
CEUS: Contrast-enhanced ultrasound
DCE: Dynamic contrast-enhanced
DWI: Diffusion weighted imaging
FI: Flow index
MRA: Magnetic resonance angiography
MR-HIFU: Magnetic resonance-guided high intensity focused ultrasound
MRI: Magnetic resonance imaging
MRV: Magnetic resonance venography
MV: Mean velocity
PRF: Pulse repetition frequency
T1w: T1-weighted
T2w: T2-weighted
UAE: Uterine artery embolization
VI: Vascularity index
